# Focus on lens connexins

**DOI:** 10.1186/s12860-016-0116-6

**Published:** 2017-01-17

**Authors:** Viviana M. Berthoud, Anaclet Ngezahayo

**Affiliations:** 1 Department of Pediatrics, University of Chicago, Chicago, IL USA; 2 Institute of Biophysics, Leibniz University Hannover, Hannover, Germany

## Abstract

The lens is an avascular organ composed of an anterior epithelial cell layer and fiber cells that form the bulk of the organ. The lens expresses connexin43 (Cx43), connexin46 (Cx46) and connexin50 (Cx50). Epithelial Cx50 has critical roles in cell proliferation and differentiation, likely involving growth factor-dependent signaling pathways. Both Cx46 and Cx50 are crucial for lens transparency; mutations in their genes have been linked to congenital and age-related cataracts. Congenital cataract-associated connexin mutants can affect protein trafficking, stability and/or function, and the functional effects may differ between gap junction channels and hemichannels. Dominantly inherited cataracts may result from effects of the connexin mutant on its wild type isotype, the other co-expressed wild type connexin and/or its interaction with other cellular components.

## Background

Several years ago, it was hypothesized that gap junctions play an important role in the lens [1]. This was later corroborated by the presence of cataracts in mice with targeted deletion of the lens fiber cell connexins, Cx46 and Cx50, and by the linkage of cataracts to mutations in the genes encoding these connexins in humans and rodents (summarized in http://cat-map.wustl.edu/; [2]). In this review, we describe connexin channel functions in the lens and we summarize the effects induced by modification/mutation of lens fiber connexins (including alterations of other lens proteins). Channel-independent functions of lens connexins have been previously reviewed [3] and will not be considered here.

### The ocular lens

**Fig. 1:**
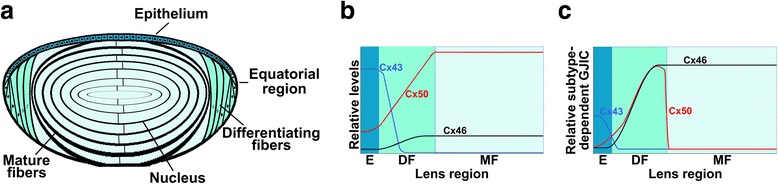
Distribution, levels and contribution of lens connexins to gap junction coupling. **a**. Diagram showing the distribution of connexin subtypes in the different regions of the lens. **b**. Diagram showing the relative amounts of Cx43, Cx46 and Cx50 in the different lens regions of the adult mouse lens. The relative values of Cx43 and Cx50 in the epithelium are based on the quinine-sensitive component of intercellular communication between mouse lens epithelial cells [111]. The relative values of Cx46 and Cx50 in mature fibers are based on analysis of the membrane proteome of the mouse lens fiber cell [13]. The relative amounts on differentiating fibers cells have been drawn partially based on the up-regulation of Cx46 and downregulation of Cx43 expressions between epithelial and mature fiber cells, and the increase in Cx50 between epithelial and fiber cells as detected by immunofluorescence. **c**. Diagram showing the relative contributions of the different connexin subtypes to gap junction intercellular coupling in cells from the different regions of the adult mouse lens. The curves summarize the data from knockout and knock-in mouse experiments [111–114]. The lens regions have been labeled E, epithelium; DF, differentiating fibers; MF, mature fibers. It is interesting to note that the contribution of Cx46 and Cx50 to gap junction coupling in fiber cells does not correlate with their protein levels

The eye lens is an avascular and transparent organ, which is responsible for focusing light onto the retina. The lens is composed of two cell types: a monolayer of epithelial cells which covers the anterior surface, and fiber cells which form the bulk of the organ (Fig. 1). Epithelial cells at the equatorial region differentiate into fiber cells through a complex process characterized by cell elongation and elimination of all cellular organelles including the nuclei. Differentiation continues to occur throughout the lifespan of the organism with newly formed fiber cells migrating over and along previous generations of fiber cells. The loss of organelles and the tight packing of the lens fiber cells contributes to minimizing light scattering [4].

### Lens connexins

The ocular lens expresses Cx43, Cx46 and Cx50 (Fig. 1). Cx43 and Cx50 are the predominant connexins expressed by epithelial cells, but these cells also express some Cx46 (mostly intracellular) [5–7]. During differentiation, expression of Cx43 is downregulated while Cx46 becomes highly expressed. Mature fiber cells express Cx46 and Cx50 [8, 9]. Transcripts for a fourth lens connexin, Cx23, have been identified in zebrafish embryo and mouse lenses [10–12]. In mouse lens fiber cells, the abundance of Cx23 protein is similar to Cx46, but lower than Cx50 [13]; however, Cx23 knockout mice have transparent lenses [14]. In the human lens, expression of Cx23 has not been reported, and it has been suggested that the Cx23 gene is inactivated in primates [12]. Because there is doubt about the expression of Cx23 in the human lens, we will not consider this connexin further.

### Functions and mechanisms supporting shared and differential roles of lens connexins

#### Shared roles

Both Cx46 and Cx50 are important for lens transparency. It is believed that the absence of either Cx46 or Cx50 alters the internal circulation system on which the avascular lens relies to maintain metabolic homeostasis. In this system, ions enter the lens at both poles along the extracellular spaces; their electrochemical gradients drive them to enter fiber cells; then, they flow back to the lens surface through gap junction channels and exit at the equator (reviewed in [15, 16]). According to this model, lens fiber gap junction channels facilitate the efflux of electrolytes from the interior of the lens. However, connexins have also been implicated in diffusion of glutathione from the outer cortex to the lens nucleus [17]. Thus, connexins may be involved in bidirectional flux (i.e., metabolites and nutrients into the center of the lens and flux of unwanted ions and byproducts to the periphery), which would be altered by absence of Cx46 or Cx50 or by mutations that modify their gap junction function. Indeed, most (but not all) of the cataract-associated connexin mutants characterized to date do not form functional gap junction channels in exogenous expression systems either because they do not traffic properly (and consequently form very few, if any, gap junction plaques) or because they form gap junction plaques that contain non-functional channels (reviewed in [18]).

#### Differential roles

The change in the expression pattern of connexins during lens differentiation suggests that connexins may have different roles in lens development and physiology. Indeed, the alterations observed in lenses lacking expression of either Cx50 or Cx46 implicate Cx50 in epithelial cell proliferation and determination of lens size [6, 19, 20], and Cx46 in Ca^2+^ homeostasis in the lens nucleus (alteration of which can lead to activation of Ca^2+^-dependent proteases) (reviewed in [21]). The role of Cx50 in determining lens size is further supported by heterozygous mice expressing different Cx50 mutants (e.g., Cx50D47A, Cx50S50P, Cx50V64A, and Cx50R205G) [7, 22–24] which have decreased lens sizes (although to different extents).

Cx50 has also been implicated in differentiation. Lenses from homozygous Cx50-null mice or homozygous Cx50G22R and Cx50S50P animals show retention of nuclei in some fiber cell layers that are deeper than normal [6, 25, 26]. In Cx50D47A mice, the alterations in degradation of nuclei and other intracellular organelles are even more pronounced, and they are observed in both homozygotes and heterozygotes [7]. These results suggest that Cx50 may mediate intercellular diffusion of second messengers required for completion of the differentiation process.

Replacement of Cx50 by Cx46 in mice (by a knock-in strategy) rescues lens transparency, but not lens size, further implying differential roles for Cx46 and Cx50 [27]. We do not yet know exactly why Cx46 cannot fully replace the function of Cx50, but this may relate to differences in the molecules permeating these channels or to differential interactions of these connexins with other lens components.

Gap junction channels formed by lens fiber cell connexins are regulated by voltage, acidification, Ca^2+^ (likely through interaction with calmodulin [28, 29]), post-translational modifications and certain chemicals (e.g., some alkanols and anesthetics, glycyrrhetinic acid, etc.). Both Cx46 and Cx50 gap junction channels are moderately cation-selective [30, 31] and have a low permeability to glutathione [17], suggesting that the differential roles rely on permeation of other molecules or on other connexin properties.

### Lens connexin function and growth factor signaling pathways

Some studies suggest that Cx50- (but not Cx46-) mediated gap junctional intercellular communication is increased by activation of the fibroblast growth factor (FGF) transduction pathways [32]. FGFs are among the growth factors that regulate epithelial lens cell proliferation and differentiation [33, 34]. Binding of FGFs to their receptors is transduced by pathways including the PI3K-AKT- and the mitogen-activated protein kinase (MAPK)-signaling pathways. Interestingly, homozygous mice with transgenic expression of a constitutively active mutant of the MAPK kinase 1 (MEK1(E)), which is upstream of MAPK, develop macrophthalmia and cataracts [35]. Deletion of Cx50 or its replacement with Cx46 decreases the MEK1(E)-induced stimulation of post-natal mitosis in the lens, reduces eye and lens growth, and delays the progression of cataracts [32].

In addition, expression of a constitutively active p110α subunit of PI3K with Cx50 or Cx46 in *Xenopus* oocytes increases Cx50 (but not Cx46) gap junctional conductance, whereas inhibition of PI3K signaling inhibits Cx50- (but not Cx46-) mediated gap junctional conductance [36]. By extrapolation, alterations in PI3K-AKT signaling would be expected to have similar effects on Cx50 in the lens. Homozygous mice with lens-specific deletion of the phosphatase and tensin homolog, *PTEN*, a protein phosphatase that can antagonize the PI3K-AKT/PKB signaling pathway, develop cataracts and show an AKT-dependent decrease in Na^+^/K^+^-ATPase activity [37]. However, the effect of *PTEN* deletion on lens fiber connexins has not been reported.

These results suggest the presence of a positive feedback circuit between FGF signaling and Cx50 gap junction function that may determine lens size. This hypothesis implies that overexpression of Cx50 accompanied with increased Cx50 gap junction channel function might result in increased lens size. However, overexpression of Cx50 in mouse primary and secondary lens fiber cells results in slowed differentiation, smaller lenses and cataracts [38]. The reason for the decrease in lens size is unknown, but it may result from the localization of some of the Cx50 in the membrane of intracellular vesicles, which may slow differentiation. Alternatively, to augment lens size the increase in Cx50 gap junction function must occur in lens epithelial cells to influence the proliferation and differentiation of these cells. However, the effect of overexpression of Cx50 in lens epithelial cells has not been tested.

### Connexin hemichannels in the normal and cataractous lens

Lens fiber cell connexins can form functional hemichannels. Rat Cx46 was the first cloned connexin shown to form functional gap junction hemichannels when expressed in *Xenopus* oocytes [8]. This property is shared with Cx46 orthologues from various species (e.g., human Cx46, bovine Cx44 and chicken Cx56) [39–41]. Cx50 is much less efficient at forming functional hemichannels; injection of larger amounts of RNA (>50 times) is required to detect hemichannel currents in *Xenopus* oocytes [42, 43].

Ebihara et al. showed opening of Cx46 hemichannels in isolated lens fiber cells studied at reduced or at normal extracellular Ca^2+^ concentrations [44, 45]. The opening of Cx46 hemichannels at physiological concentrations of Ca^2+^ may contribute to the Na^+^ leak conductance [45] and provide a pathway for the inward cation leak of the lens circulation model [46]. Because Cx46 hemichannels are mechanosensitive, it has been proposed that they participate in lens accommodation [47].

Although the lens apparently has mechanisms for controlled opening of wild type connexin hemichannels, they may be insufficient to prevent opening of those formed by connexin mutants with enhanced hemichannel activity. Three mutants identified in cataract patients, Cx50G46V, Cx46G143R and Cx46T19M, show increased hemichannel activity in exogenous expressions systems [48–50]. These mutants differ in their ability to form functional gap junction channels. While Cx50G46V elicits similar gap junction channel conductances to wild type Cx50 [48], Cx46G143R shows decreased gap junction channel activity [49] and Cx46T19M does not form functional gap junction channels [50]. Reduced hemichannel function may be pathologic. Cataract-linked connexin mutants (Cx50S276F and Cx50V44A) show decreased hemichannel function and have differential effects on gap junction channel function; Cx50S276F does not form functional gap junction channels [51], whereas Cx50V44A (expressed as a GFP fusion protein) forms functional gap junction channels [52]. However, the signaling pathways by which altered hemichannel activity leads to lens disease have not been identified.

### Can the functional defects of connexin mutants be predicted?

**Fig. 2:**
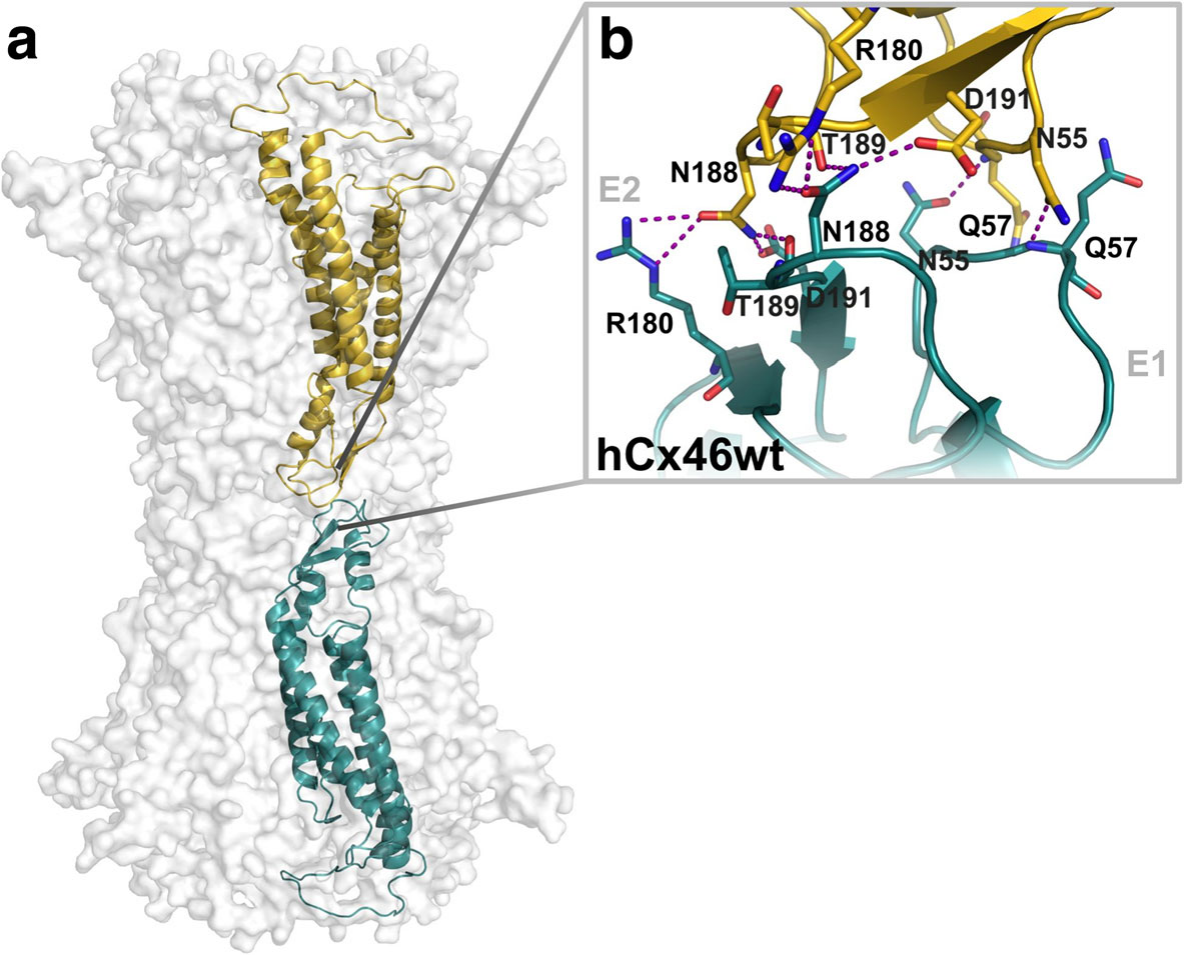
Docking of Cx46 connexons from adjacent cells. **a**. Homology model of the Cx46 gap junction channel based on the crystal structure of Cx26. The docking connexons are given in transparent surface representation. The interacting connexins of each connexon are shown as ribbons in *gold* and *cyan*, respectively. **b**. The network of hydrogen bonds between the amino acid residues of the interacting extracellular loops E1 and E2 are shown (*dashed lines*). (Reprinted with permission from [57])

The effects of mutations on function may sometimes be predicted by alignment of the lens connexins with Cx26 and inferences based on the crystal structure of the Cx26 gap junction channel [53, 54]. The Cx26 crystal structure shows that the N-termini of the six connexins in a hemichannel form a funnel that restricts the diameter at the entrance of the pore. D2 is one of the N-terminal amino acids that lines the surface of the Cx26 pore funnel forming a ring of negatively charged side chains [53, 54]. Modeling rat Cx46 based on the Cx26 crystal structure predicts that mutation of the corresponding amino acid in Cx46 (D3) to tyrosine (a bulkier polar amino acid) results in closure or partial closure of the hemichannel in the absence of applied voltage [55]. Indeed, mutation of D3 in human or rat Cx46 to tyrosine (Cx46D3Y) results in mutants with greatly reduced (or no) hemichannel activity [55, 56]. The Cx26 model has been recently used to interpret the behavior of Cx46N188T, a mutant that forms functional hemichannels, but not gap junction channels in HeLa cells [57]. Residue N188 in Cx46 corresponds to N176 of Cx26. This residue is involved in docking of Cx26 connexons through formation of hydrogen bonds between N176 from a connexon protomer in one cell and K168, T177, and D179 in the counterpart protomer of the connexon in the adjacent cell; the corresponding residues in Cx46 are R180, T189, and D191 (Fig. 2). Thus, replacement of N188 by T in Cx46 would suppress formation of the hydrogen bonds required for connexon docking and thereby abolish gap junction function.

The crystal structure of Cx26 may also be useful in predicting conformational changes affecting protein folding in some mutants and trafficking (by calculating the surface electrostatic potential [58]). However, it may not be able to predict mutations affecting internalization or degradation.

### Post-translational modifications of lens fiber connexins

The roles of connexins in the lens are also affected by post-translational modifications, which may vary in the different regions of the lens and have different functional effects. Protein cleavage and phosphorylation are considered the most common among the post-translational modifications that can alter channel function of lens connexins.

#### Cleavage

Truncation of the C-terminus has been associated with fiber cell maturation [59]. Cleaved connexins are preferentially found in the lens nucleus [59–63], where gap junction plaques are mostly devoid of cholesterol and contain crystalline-packed connexons [64]. Several cleaved forms of Cx46 (with proteolysis in any of its cytoplasmic domains) and Cx50 (with cleavage limited to the C-terminus) have been identified in bovine lenses by mass spectrometry [63], suggesting that these connexins are substrates for more than one protease. Truncation of Cx46 in the distal C-terminus in the lens cortex and closer to the fourth transmembrane domain in the lens nucleus [63] suggests that the protein goes through sequential and progressive stages of cleavage as cells grow older.

It has been postulated that cleavage of the connexin’s C-terminus in mature fiber cells represents an adaptation to maintain functional coupling in the lens nucleus, which has a more acidic pH than the lens cortex [65]. Indeed, truncation of the mouse Cx46 C-terminus shifts the pKa for gap junctional conductance to more acidic values, but does not eliminate pH sensitivity [66]. However, conflicting results have been reported for the pH sensitivity of truncated Cx50 gap junction channels, varying from similar to that of full-length Cx50 to reduced or complete loss of pH sensitivity [67–70]. The reason for these discrepancies is not clear.

Expression of cleaved forms of Cx46 or Cx50 also decreases total junctional conductance [66, 69, 70], alters localization of the protein to gap junction plaques and affects intercellular transfer of gap junction tracers [71, 72]. The extent of truncation required to abolish gap junction intercellular communication depends on the connexin subtype and varies in different studies.

Deletion of the last amino acid of Cx50 (that is important for its interaction with ZO-1; [73]) results in complete loss of gap junction plaque localization and intercellular transfer of Neurobiotin in HeLa cells [71]. Homozygous knock-in mice expressing this Cx50 deletion mutant have smaller lenses with nuclear cataracts, similar to the phenotype of Cx50-null mice [71]. More recently, Wang et al. have suggested that the distal truncation of Cx45.6 (the chicken ortholog of Cx50) protects lens cells against UV radiation [74].

Deletion of the last amino acid of rat Cx46 leads to a major decrease (but not loss) of localization of the protein at gap junctional plaques and of intercellular coupling [71]. More proximal deletions of rat Cx46 abolish intercellular transfer of gap junction tracers [71, 72]. In addition, truncation of rat Cx46 after amino acid 284 strongly reduces vesicle budding from gap junction plaques and formation of annular gap junctions, suggesting that the C-terminus modulates the rate of removal of Cx46 from gap junction plaques [72].

#### Phosphorylation

All lens connexins are phosphoproteins. Cx43 is phosphorylated at many different sites (reviewed in [75]). Phosphorylation of Cx43 has been implicated in trafficking to and from the plasma membrane, channel function and degradation (reviewed in [75]). Many phosphorylated sites in bovine lens Cx46 and Cx50 have been detected by mass spectrometry [63, 76]. Phosphorylation of lens fiber connexins has been implicated in regulation of turnover (or stability) and intercellular coupling [77–82]. Three sites involved in these processes have been identified. Phosphorylation of Cx45.6 by casein kinase II at S364 leads to increased turnover of the protein [81], whereas phosphorylation by protein kinase A at S395 has been implicated in increased channel permeability to Lucifer yellow [82]. Protein kinase C (PKC)-dependent phosphorylation of S118 in the intracellular loop of Cx56 correlates with decreased intercellular communication [78, 83].

Among the PKC isoforms expressed in the lens, PKCγ has received special attention, because it is expressed in lens fiber cells and it is activated by the phorbol ester 12-O-tetradecanoyl-13 acetate (TPA) [83–85]. Its presence seems essential for phosphorylation of Cx50 in serines and threonines, because in PKCγ knockout mouse lenses Cx50 lacks phosphorylation in these amino acid residues [86]. These lenses show structural changes in cortical fiber cell gap junctions [86], increased gap junction conductance in differentiating and mature fiber cells [87] and, unlike wild type lenses, their gap junctions remain permeable to Lucifer yellow after treatment with TPA [86]. Lenses from PKCγ knockout mice show increased levels of Cx43 (but no changes in total Cx46 or Cx50); Cx43 is present not only in the epithelium but also in differentiating fiber cells [87]. TPA-induced activation of PKC causes a proteasome-dependent decrease in Cx43 in epithelial cells that correlates with an increase in both mRNA and protein levels of Cx46 [88]. Overexpression of Cx46 leads to a proteasome-dependent decrease in Cx43 protein levels in these cells. The exact mechanism underlying these changes is not completely understood.

#### Ubiquitylation

Ubiquitylation is another post-translational modification that has been demonstrated for Cx43 and Cx45.6 [89, 90]. Transgenic expression of a mutant ubiquitin (K6W) that is conjugation competent but proteolytically incompetent in mice leads to cataracts that are associated with an increase in Cx43, a decrease in Cx46 and in intercellular coupling between fiber cells in the lens nucleus, and accumulation of calcium in the lens core (likely as a result of decreased gap junction activity) [91]. Since overexpression of K6W ubiquitin slows epithelial cell proliferation and differentiation [92], the changes in Cx43 and Cx46 levels may result at least in part from altered epithelial-to-fiber cell differentiation.

Different post-translational modifications can influence each other. For example, the caspase-3-dependent cleavage of Cx45.6 is inhibited by casein kinase II-mediated phosphorylation of Ser364 [93]. Post-translational modifications of connexins are tightly regulated, regionalized and/or progressive and help to maintain lens transparency. Therefore, mutations that alter or create novel recognition sites for phosphorylation, cleavage, ubiquitylation or other post-translational modifications may affect connexin function and lead to cataracts.

### Possible mechanisms for the inheritance pattern of cataracts

**Fig. 3:**
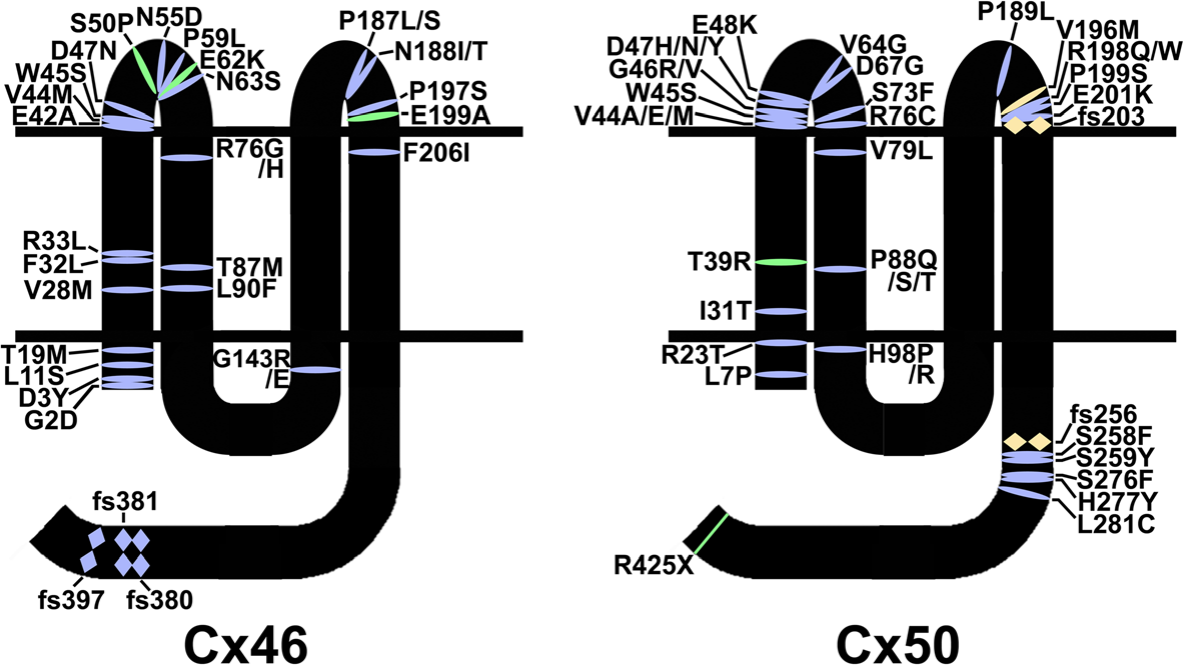
Depiction of cataract mutants in human Cx46 and Cx50. Diagrams show the membrane topology of Cx46 and Cx50 and the localization of Cx46 and Cx50 mutants found in patients with non-syndromic and syndromic cataracts. The diagrams do not include mutations predisposing to age-related cataracts. Missense mutations are represented by a fusiform shape; frame-shift mutations (fs) are depicted by double rhombi; and early termination mutations by rectangles. Cx46 and Cx50 mutants with autosomal dominant inheritance are represented in light *blue-purple*. Mutants with recessive inheritance are shown in light *golden yellow* and mutants with an unassigned or sporadic inheritance pattern are shown in *green*. For different missense mutations at the same amino acid residue, the color coding depicts a known inheritance pattern (even if it is unassigned for the other missense mutations at that site). The Cx50I247M mutant has not been depicted, because it may be a polymorphism. These diagrams are based on the information available in Cat-Map ([2]; http://cat-map.wustl.edu/)

Many mutations of Cx46 and Cx50 have been linked to congenital cataracts (summarized in Fig. 3). Most Cx46 and Cx50 gene variants are inherited as autosomal dominant traits, but some of them have been linked to recessive and age-related cataracts in humans.

#### Congenital cataracts

Considering that many of the cataract-associated mutants characterized in exogenous expression systems show loss of function (and only some of them have a dominant negative effect on co-expressed wild type connexins) and that mice with half of the Cx46 or Cx50 levels (heterozygous null mice) do not develop cataracts [19, 94], it is intriguing that most Cx46 and Cx50 mutants cause dominant cataracts. Studies on some mouse lines carrying a cataract-linked Cx50 or Cx46 mutation have revealed alterations in the co-expressed connexin. Homozygous mice for Cx50G22R show altered distribution and reduced phosphorylation of Cx46 [95]. In Cx50D47A and Cx46fs380 mice, heterozygous and homozygous lenses show a severe reduction in the levels of the connexin (mutant and wild type) and a significant but milder reduction in the co-expressed lens fiber connexin [7, 96]. Whether the change in the co-expressed connexin is due to direct interaction with the mutant or an indirect effect (e.g., the mutation affects interactions with other lens components necessary for normal behavior of the co-expressed connexin) remains to be determined. Levels of spliced *Xbp-1* transcripts in mouse lenses expressing Cx50G22R (which causes autosomal semi-dominant cataracts) are elevated in newborn heterozygotes and at 2 months of age in heterozygotes and homozygotes [26]. Cx50D47A induces ER stress, triggering activation of the PERK-ATF4 pathway [97]. These results suggest that signaling pathways of the unfolded protein response may contribute to cataract. Thus, alterations in the co-expressed wild type connexin or induction of ER stress could explain the dominant inheritance.

As a corollary, a recessive inheritance pattern would imply that the mutant does not affect the co-expressed connexin and/or the interactions with other lens components that may affect lens transparency. Characterization of one of such mutant, Cx50fs (or fs256 as termed in Fig. 3), revealed that it is actively degraded through endoplasmic reticulum-associated degradation [98].

#### Age-related cataracts

Polymorphisms in the intronic region of the Cx50 gene and a C-to-G transversion at position −39 (c. −39C > G) in the Cx46 gene may be associated with age-related cataracts in the Chinese population [99, 100]. The exact mechanism by which these mutations predispose people to age-related cataracts is unclear. However, considering that the encoded protein is normal and that a 50% decrease in Cx46 or Cx50 in mouse lenses does not affect transparency, one could speculate that mutations in the non-coding regions may affect the level of expression of the connexin to a value that is insufficient to cause cataracts early in life but the sustained alteration over long periods of time becomes more significant and leads to opacities.

A slight association between some haplotype variations in the coding region of the Cx46 and Cx50 genes (Cx46V139M and Cx50V275I) and development of age-related cataract in the Chinese population has been reported [100]. In silico analysis predicts that these mutations are benign [100]. These mutants have not been tested for effects on connexin function. If they have an effect, and because of the age of appearance of cataracts, they would be expected to have a mild effect on channel function, alterations in post-translational modifications or interactions with other lens components. This raises the possibility that in the potential case of homozygosity some of them could lead to cataracts earlier in life.

### Effects of targeted deletion or expression of mutants of other proteins on lens connexins

**Fig. 4:**
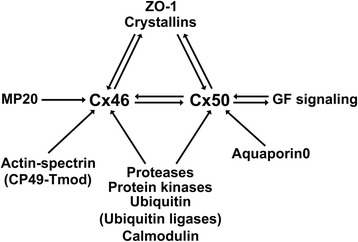
Interaction of fiber cell connexins with other lens components. The diagram depicts proteins which either interact with Cx46 and/or Cx50, or are affected by mutations in Cx46 or Cx50, or whose mutations affect Cx46 or Cx50 (as described in the text). The interaction of Cx50 with aquaporin0 is based on the report by Yu et al. [115]

Because connexin molecules are involved in networks of interaction and signaling molecules, alterations in other lens components could affect levels, distribution and/or function of connexins. This has been evaluated in some mouse models with null or missense mutations of other lens proteins including γ-crystallins, protein kinases, cytoskeletal proteins, MP20, etc. A diagram of lens proteins that interact with Cx46 and Cx50 is shown in Fig. 4. We will review some of these studies.

#### γ-Crystallins

Crystallins are the predominant cytoplasmic proteins that help maintain the proper refractive properties of the lens. Changes in their levels, solubility or abundance of cleaved/modified forms have been associated with cataracts. Many of the mouse models of cataract with targeted deletion of Cx46 or Cx50 or expressing a mutant of these connexins show alterations in lens crystallins [7, 19, 94, 96, 101]. In turn, heterozygous and homozygous mice expressing the V76D γD-crystallin mutant [102] and 10-day (but not 1-day) old homozygous S11R γB-crystallin mice [103] show decreased levels of Cx46 and Cx50. How mutations in γ-crystallins, which are synthesized during differentiation and maturation of lens fiber cells, lead to decreased connexin levels is unknown, but it has been suggested that it may relate to the disruption of membrane-cytoskeleton structures of inner fiber cells in the case of homozygous S11R γB-crystallin mice and to incomplete fiber cell differentiation in mice expressing V76D γD-crystallin [102, 103].

#### Cytoskeleton

Lens gap junction plaques rest in lacunae of the membrane-associated actin-spectrin network [104]. Disruption of this network by deletion of both phakinin (also known as CP49) and tropomodulin in mice leads to a decrease in the size of Cx46-containing gap junction plaques in differentiating fiber cells without changing the total levels of this connexin [104]. These lenses do not show cataracts at 2 months of age, but show increased light scattering and decreased coupling conductance [104, 105]. It has been suggested that disruption of the actin-spectrin network may interfere with formation or stability of large Cx46 gap junction plaques [104].

#### MP20

Lim2 (also known as MP20), a claudin-like protein, is the second most abundant integral membrane protein in the lens [13]. Its function is not clear, but it may have an adhesive role, because it is inserted in the plasma membrane in a region of the lens that is impenetrable to extracellular tracers [106]. Deletion of *Lim2* decreases Cx46 levels by ~50% in the core of the lens, whereas the levels are similar to wild type mice in the cortex [107]. These changes are associated with ~55% decrease in cell-cell coupling in differentiating and mature fibers, suggesting that the decrease in intercellular coupling in the lens core may result from reduced Cx46 levels, whereas that in the outer cell layers may ensue from absence of cell fusions [107, 108].

Collectively, these results attest to the multiple effects that can be caused by alterations in one particular lens component. While some of these are direct, others are considered secondary or indirect to such alteration.

## Conclusions

In conclusion, lens gap junction channels and connexin hemichannels are important for lens homeostasis, function and transparency. These channels have connexin subtype-specific properties and regulation. They participate in intercellular signaling via gap junction channels and may contribute to autocrine/paracrine signaling through hemichannels. At the same time, connexins can be substrates for the effectors of the signaling pathways and undergo post-translational modifications that may alter their function. Depending on the functional effect induced, this could represent a positive or a negative feedback circuit.

The effects of expression of a lens connexin mutant in the mouse on other lens components suggest that lens connexins are involved in networks of protein interactions and signaling pathways. This raises the possibility that some connexin mutants with minor (or no) effects on connexin channel function (as assessed in exogenous expression systems) may lead to cataracts by affecting other aspects of the connexin functions in the lens (e.g., the interaction with other lens proteins).

With the advent of in vitro cell culture systems that mimic lens fiber cell differentiation starting from embryonic stem cells [109, 110], it may be possible to investigate further the role of wild type connexins in lens development and differentiation and the regulation of connexin functions by different growth factors and signaling pathways at different stages of cell differentiation. Since these lentoid-forming experimental systems may be suitable for genetic manipulation, they could also be used to study the temporal course of the changes induced by altering levels or function of fiber cell connexins on other lens components as well as the mechanisms by which mutant connexins lead to cataracts as long as they faithfully recapitulate what happens in the lens in vivo.
